# Validation of the BOADICEA model in a prospective cohort of *BRCA1/2* pathogenic variant carriers

**DOI:** 10.1136/jmg-2024-109943

**Published:** 2024-06-04

**Authors:** Xin Yang, Thea M Mooij, Goska Leslie, Lorenzo Ficorella, Nadine Andrieu, Karin Kast, Christian F. Singer, Anna Jakubowska, Carla H van Gils, Yen Y Tan, Christoph Engel, Muriel A Adank, Christi J van Asperen, Margreet G E M Ausems, Pascaline Berthet, EMBRACE collaborators, Margriet J Collee, Jackie A Cook, Jacqueline Eason, Karin Y van Spaendonck-Zwarts, D. Gareth Evans, Encarna B Gómez García, Helen Hanson, Louise Izatt, Zoe Kemp, Fiona Lalloo, Christine Lasset, Fabienne Lesueur, Hannah Musgrave, Sophie Nambot, Catherine Noguès, Jan C Oosterwijk, Dominique Stoppa-lyonnet, Marc Tischkowitz, Vishakha Tripathi, Marijke R Wevers, Emily Zhao, Flora E van Leeuwen, Marjanka K Schmidt, Douglas F Easton, Matti A Rookus, Antonis C Antoniou

**Affiliations:** 1 Centre for Cancer Genetic Epidemiology, Department of Public Health and Primary Care, University of Cambridge, Strangeways Research Laboratory, Cambridge, UK; 2 Department of Epidemiology, The Netherlands Cancer Institute, Amsterdam, The Netherlands; 3 INSERM U900, Paris, France; 4 Institut Curie, Paris, France; 5 Mines ParisTech, Fontainebleau, France; 6 PSL Research University, Paris, France; 7 Center for Hereditary Breast and Ovarian Cancer, Center for Integrated Oncology (CIO), Medical Faculty, University Hospital Cologne, Cologne, Germany; 8 Department of OB/GYN and Comprehensive Cancer Center, Medical University of Vienna, Vienna, Austria; 9 Department of Genetics and Pathology, Pomeranian Medical University, Szczecin, Poland; 10 Independent Laboratory of Molecular Biology and Genetic Diagnostics, Pomeranian Medical University, Szczecin, Poland; 11 Julius Center for Health Sciences and Primary Care, University Medical Center Utrecht, Utrecht, The Netherlands; 12 Institute for Medical Informatics, Statistics and Epidemiology, University of Leipzig, Leipzig, Germany; 13 Department of Clinical Genetics, The Netherlands Cancer Institute, Antoni van Leeuwenhoek Hospital, Amsterdam, The Netherlands; 14 Department of Clinical Genetics, Leiden University Medical Center, Leiden, The Netherlands; 15 Devision Laboratories, Pharmacy and Biomedical Genetics, Department of Genetics, University Medical Centre Utrecht, Utrecht, The Netherlands; 16 Oncogénétique Département de Biopathologie, Centre François Baclesse, Caen, France; 17 Department of Clinical Genetics, Erasmus Medical Center, Rotterdam, The Netherlands; 18 Sheffield Clinical Genetics Service, Scheffield Children's Hospital, Sheffield, UK; 19 Nottingham Clinical Genetics Service, Nottingham University Hospitals NHS Trust, Nottingham, UK; 20 Department of Human Genetics, Amsterdam University Medical Centres, Amsterdam, The Netherlands; 21 The Prevent Breast Cancer Research Unit, The Nightingale Centre, Manchester University NHS Foundation Trust, Manchester, UK; 22 Genomic Medicine, Division of Evolution and Genomic Sciences, The University of Manchester, St Mary’s Hospital, Manchester University NHS Foundation Trust, Manchester, UK; 23 Manchester Breast Centre, Oglesby Cancer Research Centre, The Christie, University of Manchester, Manchester, UK; 24 Department of Clinical Genetics, Maastricht University, Maastricht, The Netherlands; 25 South West Thames Regional Genetics Service, St George’s University Hospitals NHS Foundation Trust, London, UK; 26 Department of Clinical Genetics, Guy's and St Thomas' NHS Foundation Trust, London, UK; 27 Department of Cancer Genetics, Royal Marsden Hospital, NHS Trust, London, UK; 28 Clinical Genetics Service, Manchester Centre for Genomic Medicine, Manchester University Hospitals Foundation Trust, Manchester, UK; 29 Université Claude Bernard Lyon 1, Villeurbanne, France; 30 CNRS UMR 5558, Lyon, France; 31 Centre Léon Bérard, Unité de Prévention et Epidémiologie Génétique, Lyon, France; 32 Yorkshire Regional Genetics Service, Leeds Teaching Hospitals NHS Trust, Leeds, UK; 33 Centre de Génétique et Centre de Référence Anomalies du Développement et Syndromes Malformatifs, CHU de Dijon, Hôpital d’Enfants, Dijon, France; 34 Centre de Lutte contre le Cancer Georges François Leclerc, Dijon, France; 35 Département d’Anticipation et de Suivi des Cancers, Oncogénétique Clinique, Institut Paoli Calmettes, Marseille, France; 36 Aix Marseille Univ, INSERM, IRD, SESSTIM, Marseille, France; 37 Department of Genetics, University of Groningen, University Medical Center, Groningen, The Netherlands; 38 Institut Curie, Service de Génétique, Paris, France; 39 Université Paris CIté, Paris, France; 40 INSERM U830, Paris, France; 41 Department of Medical Genetics, National Institute for Health Research Cambridge Biomedical Research Centre, University of Cambridge, Cambridge, UK; 42 Clinical Genetics Service, Guy's and St Thomas' NHS Foundation Trust, London, UK; 43 Department of Clinical Genetics, Radboud University Medical Center, Nijmegen, The Netherlands; 44 Division of Molecular Pathology and Division of Psychosocial Research and Epidemiology, The Netherlands Cancer Institute, Amsterdam, The Netherlands; 45 Centre for Cancer Genetic Epidemiology, Department of Oncology, University of Cambridge, Cambridge, UK

**Keywords:** Public Health, Genetic Counseling, Early Diagnosis, Women's Health

## Abstract

**Background:**

No validation has been conducted for the BOADICEA multifactorial breast cancer risk prediction model specifically in *BRCA1/2* pathogenic variant (PV) carriers to date. Here, we evaluated the performance of BOADICEA in predicting 5-year breast cancer risks in a prospective cohort of *BRCA1/2* PV carriers ascertained through clinical genetic centres.

**Methods:**

We evaluated the model calibration and discriminatory ability in the prospective TRANsIBCCS cohort study comprising 1614 *BRCA1* and 1365 *BRCA2* PV carriers (209 incident cases). Study participants had lifestyle, reproductive, hormonal, anthropometric risk factor information, a polygenic risk score based on 313 SNPs and family history information.

**Results:**

The full multifactorial model considering family history together with all other risk factors was well calibrated overall (E/O=1.07, 95% CI: 0.92 to 1.24) and in quintiles of predicted risk. Discrimination was maximised when all risk factors were considered (Harrell’s C-index=0.70, 95% CI: 0.67 to 0.74; area under the curve=0.79, 95% CI: 0.76 to 0.82). The model performance was similar when evaluated separately in *BRCA1* or *BRCA2* PV carriers. The full model identified 5.8%, 12.9% and 24.0% of *BRCA1/2* PV carriers with 5-year breast cancer risks of <1.65%, <3% and <5%, respectively, risk thresholds commonly used for different management and risk-reduction options.

**Conclusion:**

BOADICEA may be used to aid personalised cancer risk management and decision-making for *BRCA1* and *BRCA2* PV carriers. It is implemented in the free-access CanRisk tool (https://www.canrisk.org/).

**WHAT IS ALREADY KNOWN ON THIS TOPIC**

- No study has assessed the clinical validity of the multifactorial BOADICEA model for predicting future breast cancer risks specifically for *BRCA1/2* pathogenic variant (PV) carriers.

**WHAT THIS STUDY ADDS**

- This is the first study to validate the BOADICEA model based on the joint effects of questionnaire-based risk factors (QRFs), a polygenic risk score (PRS) based on 313 SNPs and cancer family history information on *BRCA1/2* PV carriers ascertained through clinical genetic centres. The model is well calibrated and discriminated well in both *BRCA1* and *BRCA2* carriers. The inclusion of family history, alongside QRFs and the PRS, in predicting cancer risks for PV carriers in clinical genetics settings can improve the calibration within individual risk categories and can result in clinically meaningful levels of breast cancer risk stratification.

**HOW THIS STUDY MIGHT AFFECT RESEARCH, PRACTICE OR POLICY**

- BOADICEA is freely available via the CanRisk tool (www.canrisk.org). Rather than relying solely on average published penetrance estimates commonly used in genetic clinics for counselling of *BRCA1/2* PV carriers, BOADICEA offers more personalised breast cancer risks. This can facilitate informed decision-making regarding the clinical management of breast cancer risk, including considerations for surveillance and the timing of risk-reducing surgery.

## Introduction

Women with pathogenic variants (PVs) in *BRCA1* and *BRCA2* (henceforth called ‘PV carriers’) are at high risk of developing breast cancer (BC) and ovarian cancer.^1^ However, BC risks for PV carriers vary by family history (FH) and by other genetic, lifestyle, hormonal and reproductive factors which can result in variability in the individualised BC risk assessment.^2–5^ Providing more personalised BC risks will enable informed decision-making for the clinical management of BC risk, for example, opting for bilateral risk-reducing mastectomy and its timing.

The BOADICEA model, implemented in the CanRisk tool (https://www.canrisk.org/), predicts the risk of developing BC by considering the combined effects of rare genetic variants in *BRCA1*, *BRCA2*, *PALB2*, *CHEK2*, *ATM*, *RAD51C*, *RAD51D* and *BARD1*, a polygenic risk score (PRS), FH, mammographic density (MD) and questionnaire-based risk factors (QRFs) including hormonal, lifestyle and reproductive factors.^6 7^ Previous validation studies in independent prospective cohorts have shown that the model is well calibrated and provides good discrimination in the general population.^8–10^ However, the model performance has not been evaluated specifically in *BRCA1/2* PV carriers. Here, we evaluate the performance of BOADICEA V.6^7^ in predicting BC risks in an independent prospective cohort of *BRCA1* and *BRCA2* PV carriers.

## Methods

### Subjects

Data on 2879 *BRCA1* and 2208 *BRCA2* female PV carriers were available from the prospective TRANsIBCCS cohort study.^11^ Participants were recruited via clinical genetics centres in Germany (GC-HBOC), the UK (EMBRACE), France (GENEPSO), the Netherlands (HEBON), Austria (MUV) and Poland (IHCC) and were counselled with regard to their mutation status. All participants were heterozygotes of variants considered to be pathogenic on the basis of widely accepted criteria (ENIGMA consortium; https://enigmaconsortium.org/).

All the participants were actively followed up for cancer incidence and mortality through follow-up questionnaires. In addition, follow-up through linkage with cancer, pathology and death registries has been provided in countries where these registries are available (cancer/death registries in the Netherlands and the UK; pathology registries to collect information on preventive surgeries in the Netherlands and through medical record validation of self-reported preventive surgeries).^11^

### Censoring process

All participants were followed from age at baseline to the date of BC diagnosis (invasive or ductal carcinoma in situ (DCIS)), bilateral risk-reducing mastectomy, last follow-up, death, baseline plus 6 years or age 80 years, whichever occurred first. Only those with a BC diagnosis were considered affected. A total of 344 women were censored at bilateral prophylactic mastectomy.

### Risk prediction, model calibration and discrimination

To exclude patients with potentially prevalent but undiagnosed BC at study recruitment, we predicted the 5-year BC risks starting from the age at study entry plus 1 year. The study used the latest version of BOADICEA V.6^7^ implemented in CanRisk V.2.4 (https://canrisk.org/releases/).^12^ We evaluated the model calibration and discriminatory ability. The overall calibration was assessed by the ratio of the expected (E) to the observed (O) number of patients with incident BC during the 5-year risk prediction period.^13^ We also assessed the agreement between predicted and observed risks for each individual using the calibration slope, which was calculated by fitting a logistic regression in which the dependent variable was the observed outcome (1: affected; 0: unaffected) and the independent variable was the log odds of the predicted risks. The calibration slope assesses whether the predicted risks are too extreme or conversely too moderate especially at the high and low-risk tails and is expected to be equal to 1 if the model is perfectly calibrated. The observed and expected risks were also compared in categories by grouping the samples in quintiles of predicted risks. Discrimination was assessed by the area under the receiver operating characteristic curve (AUC) and Harrell’s C-index.^14^ To assess the risk-stratifying ability of the model, we calculated the proportions of all women who had 5-year BC risks of <1.65%, <3% or <5% which are the commonly used thresholds for discussing risk-reducing options,^15 16^ and also examined the proportion of women younger than 50 years old in the low-risk groups who may opt out of or delay the risk-reducing surgeries.

From the total of 5087 women in the entire TRANsIBCCS prospective cohort, women were selected for inclusion in the analysis if they were younger than 74 years old at study entry, if they had no history of cancer or bilateral risk-reducing mastectomy, had more than 1-year follow-up and had data on QRFs and the 313-SNP PRS^17^ (online supplemental figure 1). The 313-SNP PRS was standardised using a mean of −0.424 and SD of 0.611 as described in Mavaddat *et al*.^17^ Models were then evaluated in: (1) the cohort of 2979 women who had QRF and PRS data (cohort-1); (2) among those, a cohort of 1804 women with QRF, PRS and pedigree-based cancer FH information available (cohort-2). To allow for the possibility that inclusion in these two subcohorts is non-random with respect to the incident BC status compared with the entire TRANsIBCCS prospective cohort, sampling weights were applied to the final set of eligible women in each subcohort. The sample inclusion probabilities were computed by fitting a logistic regression model in which the outcome (inclusion or not) was dependent on the age at baseline, follow-up duration, incident BC status, the interaction between BC status, age at baseline and the interaction between BC status and the follow-up duration. These were calculated for each country separately, except for Austria, Germany and Poland which were combined due to the limited sample size. The weights were then the inverse of the fitted probabilities for each individual.

10.1136/jmg-2024-109943.supp1 Supplementary data

All the statistical analyses were performed in R V.3.6.3.^18^

## Results

A total of 2979 European ancestry *BRCA1/2* PV carriers with information on PRS and QRFs were eligible for inclusion in the analysis, of whom 209 (127 *BRCA1* and 82 *BRCA2* PV carriers) developed BC during the 5-year risk prediction period (cohort-1). Among these, 1804 women (191 with incident BC) also had pedigree-based FH (cohort-2). A detailed summary of the genetic and epidemiological characteristics of the study participants at baseline is shown in online supplemental table 1. We evaluated the model separately in cohort-1 without considering FH and in cohort-2 considering the pedigree-based FH information.

**Figure 1:**
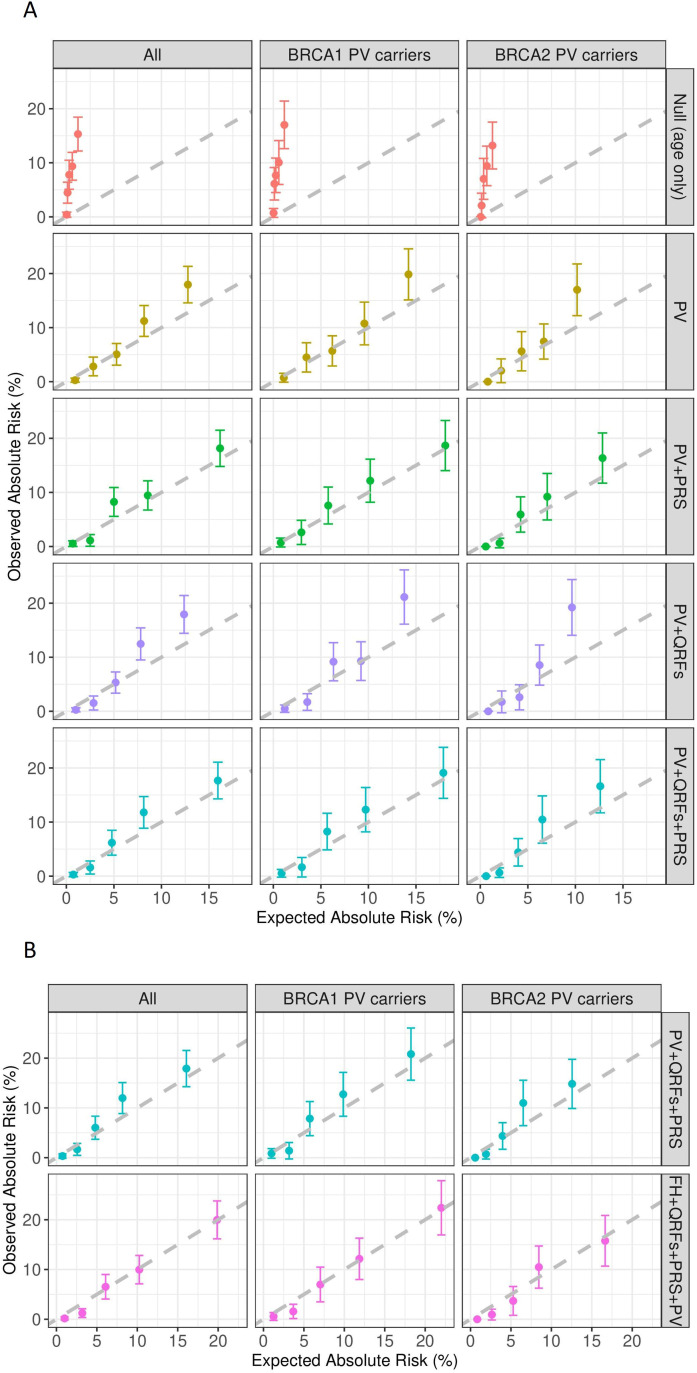
Observed and expected (E/O) 5-year breast cancer risks in quintiles of predicted risks: (A) using the cohort-1 samples (N=2979) under the models considering null (age only), PV, PV+PRS, PV+QRFs and PV+QRFs+PRS; and (B) using the cohort-2 samples with FH information (N=1804) under the models considering PV+QRFs+PRS and FH+QRFs+PRS+PV. The dashed line is the diagonal line with slope equal to 1 (corresponding to E/O ratio of 1 for each quintile). FH, family history; PRS, polygenic risk score; PV, pathogenic variant status in *BRCA1* and *BRCA2*; QRFs, questionnaire-based risk factors.

Using cohort-1, when considering *BRCA1* and *BRCA2* PV status only, or *BRCA1* and *BRCA2* PV status and QRFs, the predicted risks were underestimated (table 1), in particular for women in the higher predicted risk quintiles (figure 1A). The addition of PRS to PV status improved the calibration of the predicted risks (E/O=0.88, 95% CI: 0.76 to 1.01, calibration slope=0.95, 95% CI: 0.90 to 1.00, figure 1A). Similarly, adding PRS to the model with PV and QRF information improved calibration, but discrimination was similar (table 1).

**Table 1:** Calibration and discrimination of 5-year predicted breast cancer (BC) risks under the BOADICEA model using different risk factor combinations

Model	Category	AUC	Harrell’s C-index	E/O	Calibration slope
Using cohort-1, N=2979 including 209 incident BCs (*BRCA1*: 1614 including 127 incident BCs; *BRCA2*: 1365 including 82 incident BCs)					
Null (age only)	All women	0.70 (0.66, 0.73)	0.64 (0.59, 0.67)	0.06 (0.05, 0.07)	0.45 (0.42, 0.47)
	*BRCA1* PV carriers	0.69 (0.64, 0.74)	0.62 (0.57, 0.67)	0.05 (0.04, 0.06)	0.42 (0.39, 0.44)
	*BRCA2* PV carriers	0.72 (0.67, 0.78)	0.67 (0.61, 0.74)	0.08 (0.06, 0.10)	0.49 (0.45, 0.53)
PV	All women	0.76 (0.73, 0.80)	0.68 (0.64, 0.72)	0.80 (0.69, 0.93)	0.93 (0.88, 0.98)
	*BRCA1* PV carriers	0.75 (0.71, 0.79)	0.65 (0.59, 0.70)	0.83 (0.69, 1.00)	0.94 (0.87, 1.01)
	*BRCA2* PV carriers	0.79 (0.74, 0.84)	0.73 (0.67, 0.77)	0.75 (0.60, 0.95)	0.92 (0.85, 1.00)
PV+QRFs	All women	0.78 (0.76, 0.81)	0.69 (0.66, 0.74)	0.78 (0.67, 0.90)	0.93 (0.88, 0.98)
	*BRCA1* PV carriers	0.77 (0.73, 0.81)	0.67 (0.61, 0.72)	0.82 (0.68, 0.98)	0.94 (0.87, 1.01)
	*BRCA2* PV carriers	0.81 (0.76, 0.85)	0.74 (0.68, 0.79)	0.72 (0.57, 0.91)	0.91 (0.84, 0.99)
PV+PRS	All women	0.77 (0.73, 0.80)	0.68 (0.64, 0.71)	0.88 (0.76, 1.01)	0.95 (0.90, 1.00)
	*BRCA1* PV carriers	0.75 (0.70, 0.79)	0.66 (0.59, 0.70)	0.91 (0.75, 1.09)	0.95 (0.88, 1.02)
	*BRCA2* PV carriers	0.79 (0.74, 0.83)	0.72 (0.68, 0.78)	0.83 (0.66, 1.05)	0.95 (0.87, 1.03)
PV+QRFs+PRS	All women	0.78 (0.75, 0.81)	0.69 (0.66, 0.73)	0.86 (0.74, 0.99)	0.95 (0.89, 1.00)
	*BRCA1* PV carriers	0.76 (0.72, 0.80)	0.66 (0.62, 0.72)	0.89 (0.74, 1.07)	0.95 (0.88, 1.02)
	*BRCA2* PV carriers	0.80 (0.76, 0.84)	0.73 (0.69, 0.78)	0.80 (0.64, 1.01)	0.94 (0.86, 1.01)
Using cohort-2, N=1804 including 191 incident BCs (*BRCA1*: 1016 including 118 incident BCs; *BRCA2*: 788 including 73 incident BCs)					
PV+QRFs+PRS	All women	0.78 (0.75, 0.81)	0.69 (0.65, 0.72)	0.85 (0.74, 0.99)	0.94 (0.89, 1.00)
	*BRCA1* PV carriers	0.76 (0.72, 0.80)	0.67 (0.62, 0.72)	0.87 (0.72, 1.05)	0.95 (0.88, 1.02)
	*BRCA2* PV carriers	0.78 (0.74, 0.83)	0.72 (0.67, 0.78)	0.83 (0.65, 1.06)	0.94 (0.86, 1.02)
FH+QRFs+PRS+PV	All women	0.79 (0.76, 0.82)	0.70 (0.67, 0.74)	1.07 (0.92, 1.24)	1.06 (1.00, 1.12)
	*BRCA1* PV carriers	0.78 (0.74, 0.82)	0.69 (0.62, 0.74)	1.05 (0.87, 1.27)	1.05 (0.97, 1.13)
	*BRCA2* PV carriers	0.79 (0.75, 0.84)	0.72 (0.66, 0.77)	1.10 (0.86, 1.40)	1.07 (0.98, 1.16)

AUC, area under the receiver operating characteristic curve; FH, family history; PRS, polygenic risk score; PV, pathogenic variant status in *BRCA1* and *BRCA2*; QRFs, questionnaire-based risk factors.

Using cohort-2, we first assessed the model predictions by leaving FH out to contrast against the results in cohort-1. The model discriminatory ability and model calibration were similar to the estimates using all 2979 samples (table 1). These suggest that no bias was introduced when using the weighting cohort approach in analysing the data. After including full pedigree FH information in the model 5-year risk predictions, the model was well calibrated (overall E/O=1.07, 95% CI: 0.92 to 1.24; calibration slope=1.06, 95% CI: 1.00 to 1.12, table 1 and figure 1B). There was a small increase in the model discriminatory ability (Harrell’s C=0.70, 95% CI: 0.67 to 0.74; AUC=0.79, 95% CI: 0.76 to 0.82, table 1). The model performance was similar in *BRCA1* and *BRCA2* PV carriers (table 1 and figure 1B).

When considering all risk factors jointly, the predicted 5-year risks varied from 0.1% to 47.6%. A total of 5.8%, 12.9% and 24.0% of women had 5-year BC risks of <1.65%, <3% and <5% with a negative predictive value at the 5% risk threshold of 0.96 (95% CI: 0.96 to 0.97). 98.0% of women with a 5-year BC risk of 3% or lower, and 95.7% women (including all *BRCA1* PV carriers and 91.5% of *BRCA2* PV carriers) with a 5-year BC risk of 5% or lower were younger than 50 years old. Among women younger than 50 years old, 98.5% with 5-year risk of <5% remained unaffected during the risk prediction period. Furthermore, 78.4% of women younger than 30 years old were predicted to have 5-year risk of <5%; among them, 99.2% remained unaffected during the risk prediction period.

## Discussion

Previous validation studies have demonstrated that BOADICEA provides valid BC risks for women in the general population or women participating in screening programmes.^8–10^ Since *BRCA1/2* PVs are rare in the population, it has not been possible to assess the model performance specifically in PV carriers who are typically seen in clinical genetics.^10^ Although previous studies have indicated that multiple risk factors modify the BC risks for PV carriers,^2 19–22^ their combined effects on risk prediction^1^ have not been studied. Here, for the first time, we examined the model performance of the multifactorial BOADICEA model in predicting BC risks in *BRCA1* and *BRCA2* PV carriers seen at clinical genetics using information on PV, PRS, QRFs and FH jointly and showed that the BOADICEA is well calibrated and discriminated in this population. The results suggest that considering FH when predicting cancer risks for PV carriers seen in clinical genetics, in addition to QRFs and the PRS, can improve the calibration within individual risk categories. Given the majority of such women come from families with cancer FH, and the FH distribution in this cohort is not representative of the distribution in the general population, ignoring FH can result in some underprediction of risk among those who are at higher risk. Therefore, considering only average, published penetrance estimates for the counselling of *BRCA1/2* PV carriers typically seen in genetic clinics may underestimate BC risks—a scenario equivalent to the predictions in cohort-1, when using only *BRCA1* and *BRCA2* PV status. The analyses considered the full pedigree-based FH collected, which included third-degree or more distant relatives. When the analysis was restricted to include only first or second-degree relatives, the model performance was comparable (online supplemental table 2 and online supplemental figure 2), indicating the collection of less extensive FH may be cost-effective in clinical risk assessment.

Here, in the cohort of *BRCA1* and *BRCA2* PV carriers, the AUC of 0.79 (95% CI=0.76 to 0.82) is higher than estimates from validation studies in population-based cohorts.^8–10^ Terry *et al*, using multigenerational pedigree data from Australia, Canada and the USA,^23^ showed that a previous version of BOADICEA that considered FH and PV status only had a C-index of 0.59 and overpredicted the 10-year risk for combined *BRCA1* and *BRCA2* PV carriers in the highest quintile. However, the study used an older version of BOADICEA (V.3). Here, we used the latest model,^7 12^ and the analysis included additional risk factors (eg, QRFs and PRS). These, together with the differences in the risk prediction period, the age distributions and other cohort characteristics, make a direct comparison difficult. The present study suggests that the latest model is well calibrated across different risk categories in *BRCA1* and *BRCA2* carriers.The AUC estimates here could potentially have been overestimated because the risks for healthy women were predicted to the censoring age if they were censored within the risk prediction period. To address this, we also estimated and presented the Harrell’s C-index^14^ which considers time to event. The Harrell’s C-index yielded lower estimates than the AUC for all models. The full model that jointly considered all risk factors provided the highest discrimination as measured by Harrell’s C-index (table 1). Another potential explanation of the higher discriminatory ability observed in the current study is most likely due to the larger effect of age on BC risks for *BRCA1* and *BRCA2* PV carriers compared with the general population and the age range of study participants in this study. When only age was considered in the model, the estimated AUC in the present cohort of *BRCA1* and *BRCA2* PV carriers was 0.70 (95% CI: 0.66 to 0.73), much higher than the effect of age alone in population-based studies^10^ (figure 1A and table 1, cohort-1).

The changes in the C-index (or AUC) by the inclusion of additional risk factors on top of PV status are not significant, based on the associated CIs. This could be a consequence of the relatively small sample size. Nevertheless, the full model that includes PV status, FH, PRS and QRFs has the highest C-index. Given the high BC risks for *BRCA1/2* PV, even modest increases in the C-index can lead to changes in risk stratification.^10^ For example, when considering the half of the PV carriers with the highest predicted risks, the full model identifies 91.2% of incident BCs occurring during the prediction period. This compares with identifying 82.2% of incident BCs when only age and PV are considered. Moreover, the observed variability in the BOADICEA-predicted risks suggests that it is possible to identify *BRCA1* and *BRCA2* PV carriers with relatively low risks, in particular among women under 50 years old (or women under 30 years), who remain disease-free during the 5-year period. The results suggest that during the genetic counselling process, considering the joint effects of risk factors could be informative for decisions on the timing of risk-reducing interventions.

Analysis was repeated by censoring women diagnosed with DCIS as unaffected at the age at diagnosis (online supplemental table 3 and online supplemental figure 3). The model discriminatory ability as measured by the AUC remained similar to the overall analyses, when DCIS was considered as affected; as expected, there was some increase in the ratio of E/O cases (1.18; 95% CI: 1.01 to 1.38) and the calibration slope (1.11, 95% CI: 1.05 to 1.17) for the full model using cohort-2, suggesting some overall overprediction of risks. However, the model was still well calibrated within quintiles of predicted risk, with no significant differences between the observed and predicted risks (online supplemental figure 3).

BOADICEA does not consider the potential effect of risk-reducing salpingo-oophorectomy (RRSO) on BC risk. Censoring at RRSO resulted in some miscalibration in quintiles of predicted risk (online supplemental figure 4). Previous studies have shown that MD is also a risk factor for BC in *BRCA1* and *BRCA2* PV carriers.^24 25^ Although BOADICEA considers the effect of MD in predicting BC risks, the number of women with MD data at baseline was too small (N=794) to allow for a model assessment, which is a major limitation of the study. The number of *BRCA1* and *BRCA2* carriers was relatively small when divided by age. Nevertheless, when assessed separately by age 50 years, the full model was well calibrated in the <50 years age group. There was some overprediction in women aged 50 years or older with E/O ratio of 1.28 (95% CI: 0.96 to 1.71), but this was not significant (online supplemental figure 5 and online supplemental table 4). Larger number of carriers at older ages, with a larger number of incident cancers, will be required to assess the predicted risks with greater precision, in particular among different risk categories.^26^ BOADICEA assumes that the joint effects of *BRCA1/2* PVs with the PRS and QRFs are multiplicative on the risk scale,^6 7^ but studies suggested that deviations from the multiplicative model may exist.^2 5^ The BOADICEA model assumes an age-dependent effect of the PRS, as previously described^2^ and the present study suggests the BOADICEA assumptions provide valid risks for *BRCA1/2* PV carriers. Much larger sample sizes will be required to detect small deviations between the observed and predicted risks.

In conclusion, in the overall TRANsIBCCS prospective cohort of *BRCA1* and *BRCA2* PV carriers who were ascertained through genetic clinics, primarily on the basis of cancer FH, the multifactorial BOADICEA provided good discriminatory ability and was calibrated in predicting 5-year risks within different risk categories. The results suggest that BOADICEA may be used to aid personalised cancer risk management and decision-making for *BRCA1* and *BRCA2* PV carriers. However, the number of PV carriers by country was too small to assess differences in the predictive ability of the model by country or to assess how potential differences in data collection practices for outcomes and elective surgeries by country/study may influence the results. Future studies with much larger sample sizes of *BRCA1* and *BRCA2* PV carriers by country and with long-term follow-up should be performed to assess BOADICEA. Furthermore, it will be important to assess whether the prediction performance can be improved by using *BRCA1/2*-specific parameter estimates for the effects of the PRS and QRFs in the model.

## Data Availability

Data are available upon reasonable request. After review of the study proposal by the International BRCA1/2 Carrier Cohort Study (IBCCS) Data Access Coordinating Committee; please contact y.tan@nki.nl and mk.schmidt@nki.nl for further information.
