# Supplementary material for: Validation of the BOADICEA model in a prospective cohort of BRCA1/2 pathogenic variant carriers
Source: J Med Genet. 2024 Jun 4;61(8):803–9. doi: 10.1136/jmg-2024-109943 (PMC11287562; doi:10.1136/jmg-2024-109943)
Supplement: Supplementary data [file jmg-2024-109943supp001.pdf]

Supplementary Material

Methods

Censoring at risk-reducing salpingo-oophorectomy (RRSO)

BOADICEA does not consider the potential effect of risk-reducing salpingo-oophorectomy (RRSO) on breast cancer risk. To assess the possible impact on the results we considered RRSO as a censoring event in the analysis. This reduced the number of incident breast cancers by 48% (Table s1) and model performance estimates were associated with wide confidence intervals. Although there was an increase in the estimated AUC, there were larger deviations between the observed and expected numbers of cases in the individual quintiles of predicted risk compared to the analysis that ignored RRSO (Figure s3). The results suggest that RRSO should not be used as a censoring event when applying BOADICEA in *BRCA1/2* carriers in line with the lack of a pronounced effect of RRSO on breast cancer risk in published studies [1, 2].

Yang X, *et al. J Med Genet* 2024; 61:803–809. doi: 10.1136/jmg-2024-109943

Table s1 A summary of genetic and epidemiological characteristics of the eligible participants at baseline. Percentage was shown in women with information available.

	Healthy women	Incident BC cases[a]	Incident DCIS cases[b]
**Number of participants, N**			
**Cohort-1**	2770	186	23
***BRCA1* PV carriers**	1487	116	11
***BRCA2* PV carriers**	1283	70	12
**Cohort-2**	1613	171	20
***BRCA1* PV carriers**	898	107	11
***BRCA2* PV carriers**	715	64	9
**PRS, mean (sd)**			
	0.03 (1.04)	0.31 (1.09)	0.47 (0.73)
**Age at baseline, N (%)**			
**<30**	492 (17.8%)	17 (9.1%)	1 (4.3%)
**[30,40)**	847 (30.6%)	53(28.5%)	8 (34.8%)
**[40,50)**	710 (25.6%)	61 (32.8%)	8 (34.8%)
**[50,60)**	418 (15.1%)	37(19.9%)	5 (21.7%)
**[60,70)**	243 (8.8%)	17 (9.1%)	1 (4.3%)
**≥70**	60 (2.2%)	1 (0.5%)	0 (0.0%)
**median (IQR), years**	42 (32-50)	44 (36-52)	42 (36, 50)
**Follow-up time, years**			
**mean (sd)**	3.6 (1.4)	2.7 (1.5)	2.2 (1.3)
**Median (IQR)**	4.0 (3.0-5.0)	2.0 (1.0-4.0)	2.0 (1.0-3.0)
**Age at menarche, N (%)**			
**<11**	89 (3.5%)	9 (5.6%)	0 (0.0%)
**[11,12)**	353 (14.0%)	20 (12.5%)	2 (10.0%)
**[12,13)**	562 (22.4%)	38 (23.8%)	3 (15.0%)
**[13,14)**	612 (24.3%)	37 (23.1%)	7 (35.0%)
**[14,15)**	497 (19.8%)	34 (21.2%)	2 (10.0%)
**[15,16)**	233 (9.3%)	16 (10.0%)	5 (25.0%)
**≥16**	168 (6.7%)	6 (3.8%)	1 (5.0%)
**Missing**	256	26	3
**Menopausal status, N (%)**			
**Pre-menopausal**	1715 (61.9%)	118 (63.4%)	14 (60.9%)
**Post-menopausal**	1055 (38.1%)	68 (36.6%)	9 (39.1%)
**Age at menopause (among post-menopausal women), N (%)**			
**<40**	230 (22.9%)	9 (13.4%)	3 (33.3%)
**[40,45)**	231 (23.0%)	18 (26.9%)	2 (22.2%)
**[45,50)**	248 (24.6%)	19 (28.4%)	3 (33.3%)
**[50,55)**	257 (25.5%)	18 (26.9%)	0 (0.0%)
**≥55**	40 (4.0%)	3 (4.5%)	1 (11.1%)
**Missing**	49	1	0
**Use of hormonal replacement treatment (among post-menopausal women), N (%)**			

Yang X, *et al. J Med Genet* 2024; 61:803–809. doi: 10.1136/jmg-2024-109943

**Current estrogen only type**	97 (10.1%)	7 (11.3%)	0
**Current other type**	61 (6.4%)	4 (6.5%)	0
**Former**	169 (17.6%)	4 (6.5%)	1 (14.3%)
**Never**	631 (65.9%)	47 (75.8%)	6 (85.7%)
**Missing**	97	6	2
**Parity, N (%)**			
**0**	896 (32.4%)	51 (27.4%)	4 (17.4%)
**1**	471 (17.1%)	33 (17.7%)	4 (17.4%)
**2**	899 (32.6%)	62 (33.3%)	7 (30.4%)
**≥3**	495 (17.9%)	40 (21.5%)	8 (34.8%)
**Missing**	9	0	0
**Age at first live birth (among parous women), N (%)**			
**<20**	161 (8.7%)	11 (8.3%)	0 (0.0%)
**[20,25)**	553 (29.9%)	42 (31.6%)	8 (42.1%)
**[25,30)**	717 (38.7%)	56 (42.1%)	5 (26.3%)
**≥30**	421 (22.7%)	24 (18.0%)	6 (31.6%)
**Missing**	22	2	0
**Use of oral contraceptive, N (%)**			
**Current**	675 (25.7%)	37 (21.6%)	2 (10.0%)
**Former**	1632 (62.2%)	116 (67.8%)	17 (85.0%)
**Never**	317 (12.1%)	18 (10.5%)	1 (5.0%)
**Missing**	146	15	3
**Body Mass Index (kg/m2), N (%)**			
**<18.5**	95 (3.5%)	5 (2.7%)	1 (4.5%)
**[18.5,25)**	1561 (57.4%)	109 (59.6%)	12 (54.5%)
**[25,30)**	679 (25.0%)	50 (27.3%)	4 (18.2%)
**≥30**	382 (14.1%)	19 (10.4%)	5 (22.7%)
**Missing**	53	3	1
**Height (cm), N (%)**			
**<152.91**	112 (4.1%)	5 (2.7%)	0 (0.0%)
**[152.91, 159.65)**	372 (13.6%)	25 (13.7%)	6 (27.3%)
**[159.65, 165.96)**	914 (33.4%)	52 (28.4%)	4 (18.2%)
**[165.96, 172.70)**	824 (30.2%)	66 (36.1%)	7 (31.8%)
**≥172.70**	511 (18.7%)	35 (19.1%)	5 (22.7%)
**Missing**	37	3	1
**Alcohol consumption (g/day), N (%)**			
**<5**	1111 (43.1%)	66 (37.5%)	7 (36.8%)
**[5,15)**	1003 (39.0%)	75 (42.6%)	6 (31.6%)
**[15,25)**	272 (10.6%)	19 (10.8%)	5 (26.3%)
**[25,35)**	122 (4.7%)	9 (5.1%)	0 (0.0%)
**[35,45)**	45 (1.7%)	5 (2.8%)	1 (5.3%)
**≥45**	22 (0.9%)	2 (1.1%)	0 (0.0%)
**Missing**	195	10	4
**Risk-reducing salpingo-oophorectomy, N (%)**			
**Cohort-1:**			

**Women with RRSO before the baseline**	1070 (38.6%)	83 (44.6%)	12 (92.3%)
**Censored after the baseline**	169 (6.1%)	9 (4.8%)	1 (7.7%)
**Cohort-2:**			
**Women with RRSO before the baseline**	666 (41.3%)	74 (43.3%)	10 (90.9%)
**Censored after the baseline**	116 (7.2%)	8 (4.7%)	1 (9.1%)

[a]Incident breast cancer cases during the five-year prediction period.
[b]Incident ductal carcinoma in situ cases during the five-year prediction period.
PV: pathogenic variant; FH: family history.

Yang X, et al. J Med Genet 2024; 61:803–809. doi: 10.1136/jmg-2024-109943

Table s2: Calibration and discrimination of five-year predicted breast cancer risks using the cohort-2 samples (N=1,804) under the model considering pathogenic variant status in *BRCA1* and *BRCA2,* questionnaire-based risk factors, polygenic risk score and family history (FH). Model performance was examined by including information on all available relatives, or only first or second degree relatives.

Degrees of relatives included in the pedigree-based FH	Category	AUC	Harrell's C-index	E/O	Calibration slope
1st degree relatives only	All women	0.79 (0.76, 0.82)	0.70 (0.66, 0.74)	1.01 (0.87, 1.17)	1.03 (0.97, 1.09)
	BRCA1 carriers	0.78 (0.74, 0.82)	0.68 (0.63, 0.74)	1.01 (0.83, 1.22)	1.02 (0.95, 1.10)
	BRCA2 carriers	0.79 (0.75, 0.84)	0.72 (0.66, 0.79)	1.01 (0.79, 1.30)	1.03 (0.94, 1.12)
1st and 2nd relatives only	All women	0.79 (0.76, 0.82)	0.70 (0.66, 0.74)	1.05 (0.90, 1.22)	1.05 (0.99, 1.11)
	BRCA1 carriers	0.78 (0.74, 0.82)	0.69 (0.64, 0.73)	1.04 (0.86, 1.25)	1.04 (0.96, 1.12)
	BRCA2 carriers	0.79 (0.74, 0.84)	0.71 (0.65, 0.78)	1.07 (0.84, 1.37)	1.06 (0.97, 1.15)
Full collected pedigrees	All women	0.79 (0.76, 0.82)	0.70 (0.67, 0.74)	1.07 (0.92, 1.24)	1.06 (1.00, 1.12)
	BRCA1 carriers	0.78 (0.74, 0.82)	0.69 (0.62, 0.74)	1.05 (0.87, 1.27)	1.05 (0.97, 1.13)
	BRCA2 carriers	0.79 (0.75, 0.84)	0.72 (0.66, 0.77)	1.10 (0.86, 1.40)	1.07 (0.98, 1.16)

Yang X, *et al. J Med Genet* 2024; 61:803–809. doi: 10.1136/jmg-2024-109943

Table s3: Calibration and discrimination of five-year predicted breast cancer risks under the BOADICEA model using different risk factor combinations by censoring DCIS (ductal carcinoma in situ) as unaffected.

Model	Category	N.unaffected	N.BCs	AUC	Harrell's C-index	E/O	Calibration slope
**using cohort-1**							
PV+QRFs+PRS	All women	2793	186	0.78 (0.75, 0.81)	0.69 (0.66, 0.73)	0.94 (0.81, 1.10)	0.99 (0.93, 1.04)
	BRCA1 carriers	1498	116	0.76 (0.71, 0.80)	0.66 (0.61, 0.70)	0.96 (0.79, 1.16)	0.99 (0.91, 1.06)
	BRCA2 carriers	1295	70	0.80 (0.76, 0.85)	0.74 (0.69, 0.80)	0.91 (0.71, 1.17)	0.99 (0.91, 1.08)
**using cohort-2**							
PV+QRFs+PRS+FH	All women	1633	171	0.79 (0.76, 0.82)	0.70 (0.66, 0.74)	1.18 (1.01, 1.38)	1.11 (1.05, 1.17)
	BRCA1 carriers	909	107	0.78 (0.73, 0.82)	0.68 (0.62, 0.73)	1.15 (0.94, 1.40)	1.09 (1.01, 1.17)
	BRCA2 carriers	724	64	0.80 (0.75, 0.85)	0.73 (0.68, 0.77)	1.24 (0.95, 1.61)	1.13 (1.03, 1.23)

PV: pathogenic variant status in *BRCA1* and *BRCA2*; QRFs: questionnaire-based risk factors; PRS: polygenic risk score; FH: family history

Table s4: Calibration and discrimination of five-year predicted breast cancer risks using the cohort-2 samples (N=1,804) under the full model considering pathogenic variant status in *BRCA1* and *BRCA2,* questionnaire-based risk factors, polygenic risk score and family history by age group.

**Age**	**N.Unaffected**	**N.BCs**	**AUC**	**Harrell's C-index**	**E/O**	**Calibration slope**
< 50 years	1190	139	0.80 (0.77, 0.84)	0.72 (0.66, 0.75)	1.00 (0.84, 1.19)	1.03 (0.96, 1.09)
≥ 50 years	423	52	0.75 (0.67, 0.82)	0.64 (0.55, 0.71)	1.28 (0.96, 1.71)	1.16 (1.03, 1.29)

Yang X, *et al. J Med Genet* 2024; 61:803–809. doi: 10.1136/jmg-2024-109943

Figure s1: Consort diagram summarising the TRANsIBCCS cohort data

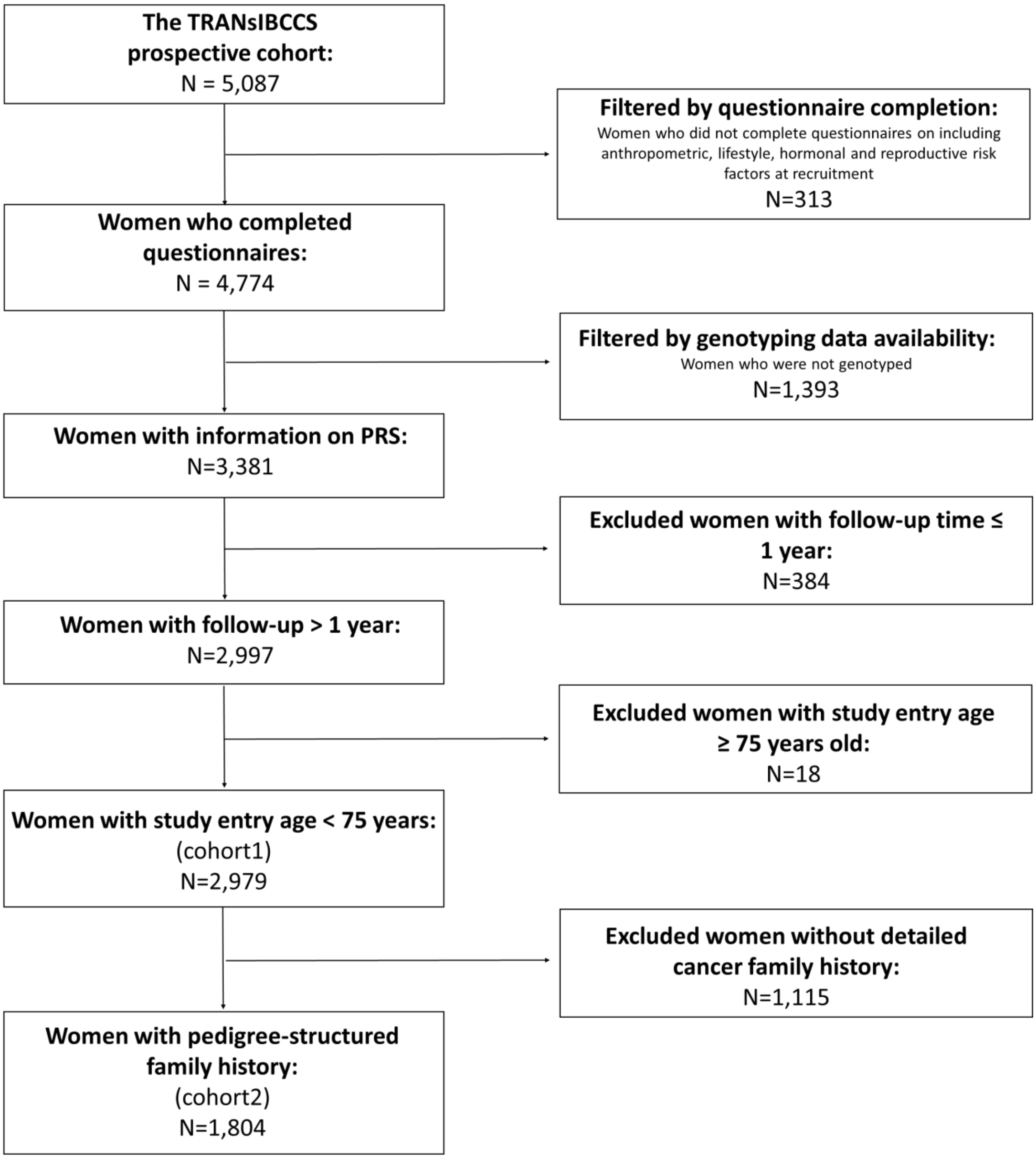

Figure s2: Observed and expected five-year breast cancer risks in quintiles of predicted risks, using the cohort-2 samples (N=1,804) under the model considering pathogenic variant status in *BRCA1* and *BRCA2,* questionnaire-based risk factors, polygenic risk score and family history. Model performance was examined by considering (a) only 1st degree relatives, (b) 1st and 2nd degree relatives and (c) the full collected pedigrees including more distant relatives. The dashed line is the diagonal line with slope equal to 1 (corresponding to E/O ratio of 1 for each quintile).

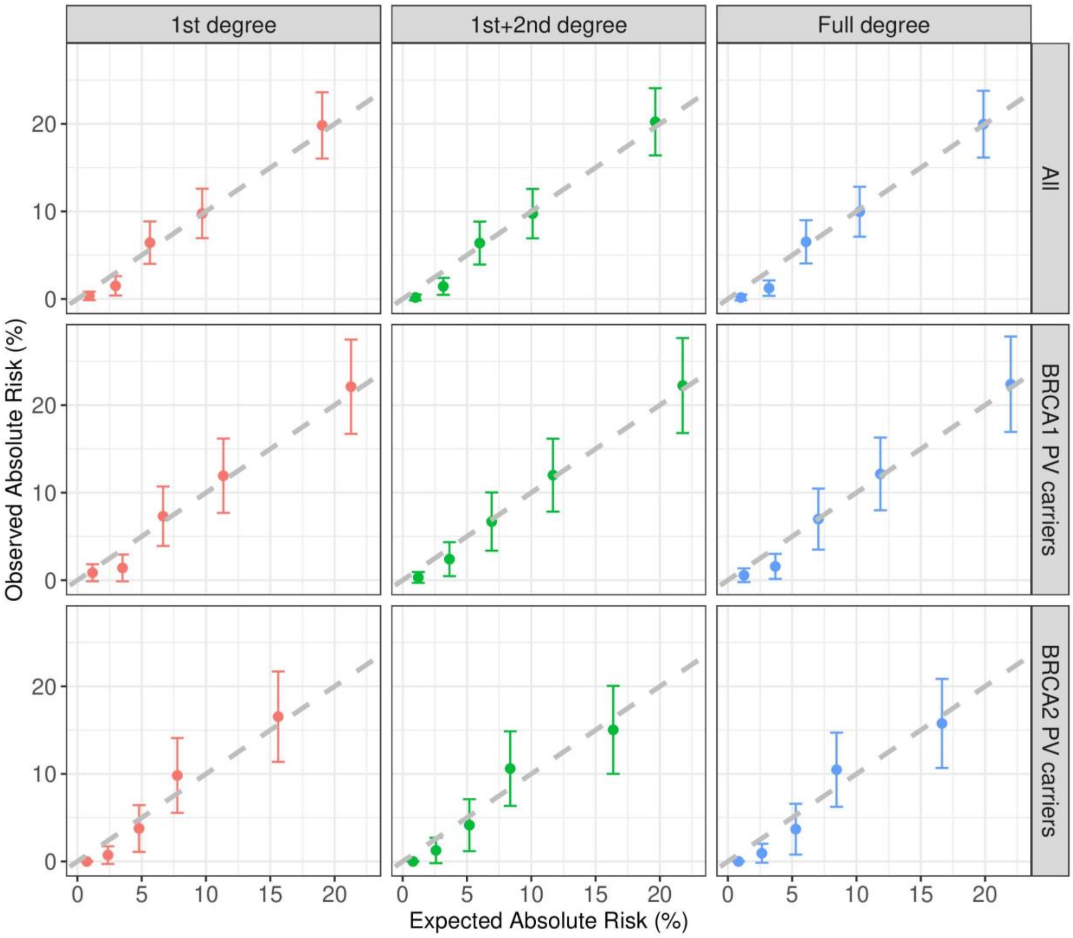

Yang X, *et al. J Med Genet* 2024; 61:803–809. doi: 10.1136/jmg-2024-109943

Figure s3: Observed and expected five-year breast cancer risks in quintiles of predicted risks, using (1) the cohort-1 samples (N=2,979) under the model considering PV, QRFs and PRS; (2) the cohort-2 samples with FH information (N=1,804) under the model considering PV, QRFs, PRS and FH by censoring DCIS (ductal carcinoma in situ) as unaffected. The dashed line is the diagonal line with slope equal to 1 (corresponding to E/O ratio of 1 for each quintile). PV: pathogenic variant status in *BRCA1* and *BRCA2*; QRFs: questionnaire-based risk factors; PRS: polygenic risk score; FH: family history.

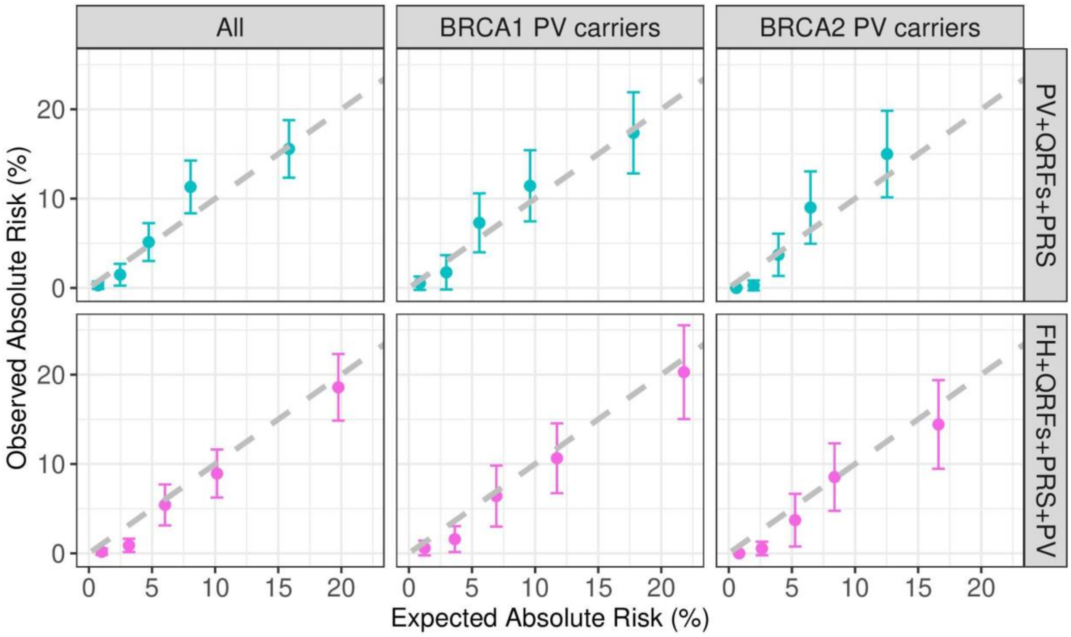

Figure s4: Observed and expected five-year breast cancer risks in quintiles of predicted risks, using the cohort-2 samples when censoring at risk-reducing salpingo-oophorectomy (N=1,054 eligible at baseline) under the model considering PV, QRFs, PRS and FH. The dashed line is the diagonal line with slope equal to 1 (corresponding to E/O ratio of 1 for each quintile). PV: pathogenic variant status in *BRCA1* and *BRCA2*; QRFs: questionnaire-based risk factors; PRS: polygenic risk score; FH: family history.

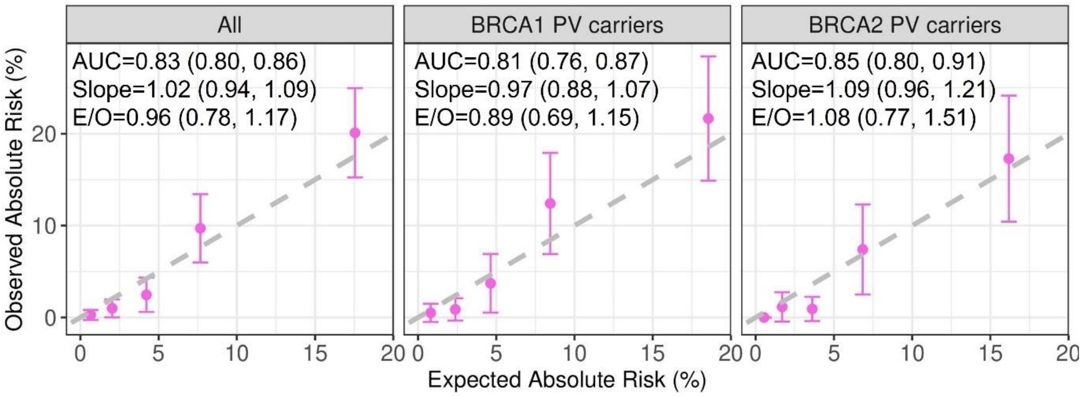

Figure s5: Observed and expected five-year breast cancer risks in quintiles of predicted risks, using the cohort-2 samples (N=1,804) under the model considering pathogenic variant status in *BRCA1* and *BRCA2*, questionnaire-based risk factors, polygenic risk score and family history by age group.

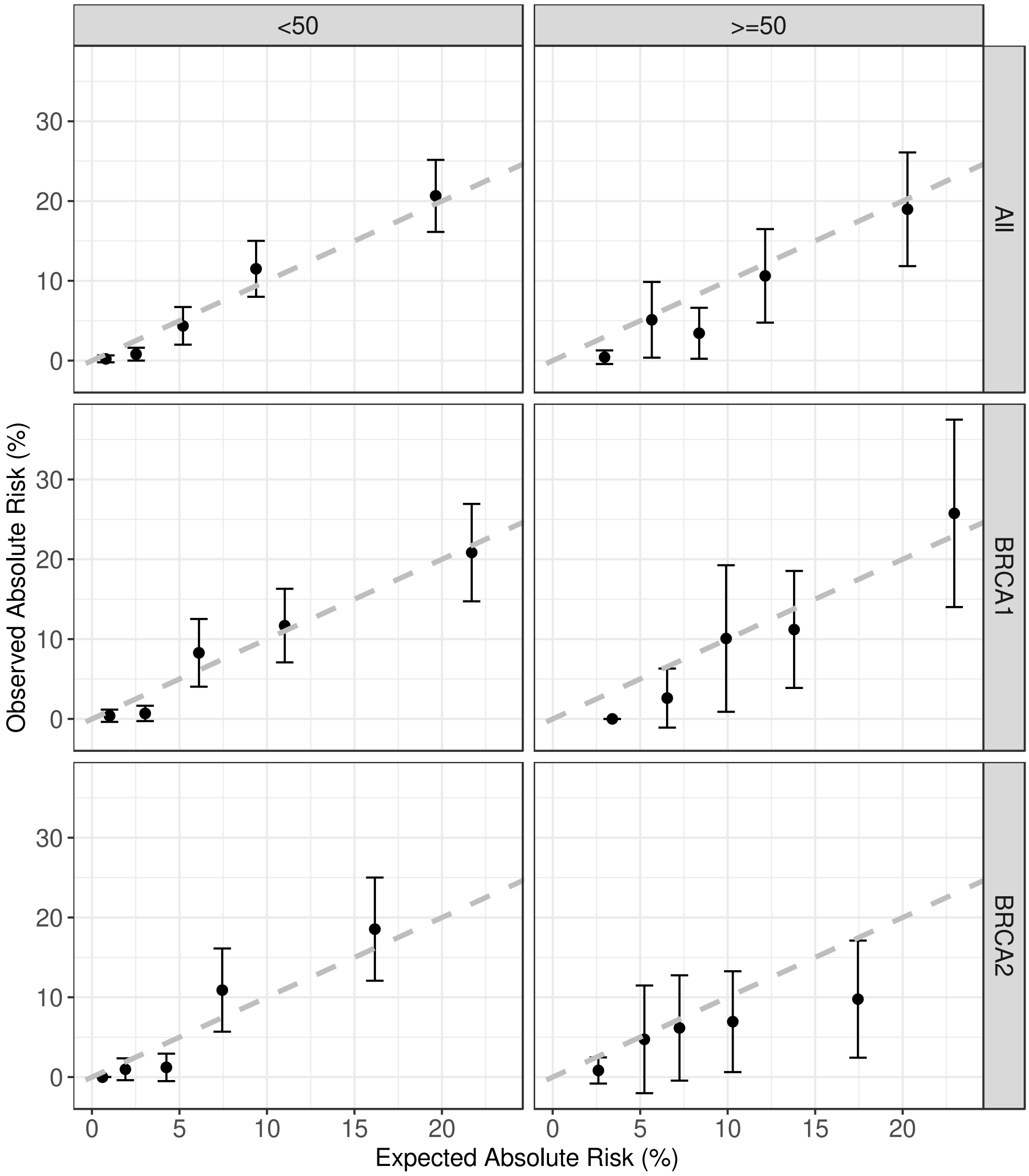

Yang X, *et al. J Med Genet* 2024; 61:803–809. doi: 10.1136/jmg-2024-109943

References:

1. Mavaddat N, Antoniou AC, Mooij TM, Hooning MJ, Heemskerk-Gerritsen BA, Genepso, Nogues C, Gauthier-Villars M, Caron O, Gesta P, Pujol P, Lortholary A, Embrace, Barrowdale D, Frost D, Evans DG, Izatt L, Adlard J, Eeles R, Brewer C, Tischkowitz M, Henderson A, Cook J, Eccles D, Hebon, van Engelen K, Mourits MJE, Ausems M, Koppert LB, Hopper JL, John EM, Chung WK, Andrulis IL, Daly MB, Buys SS, kConFab I, Benitez J, Caldes T, Jakubowska A, Simard J, Singer CF, Tan Y, Olah E, Navratilova M, Foretova L, Gerdes AM, Roos-Blom MJ, Van Leeuwen FE, Arver B, Olsson H, Schmutzler RK, Engel C, Kast K, Phillips KA, Terry MB, Milne RL, Goldgar DE, Rookus MA, Andrieu N, Easton DF, Ibccs, kConFab, Bcfr. Risk-reducing salpingo-oophorectomy, natural menopause, and breast cancer risk: an international prospective cohort of BRCA1 and BRCA2 mutation carriers. *Breast Cancer Res* 2020;**22**(1):8 doi: 10.1186/s13058-020-1247-4[published Online First: 2020/01/18].
2. Stuursma A, van der Vegt B, Jansen L, Berger LPV, Mourits MJE, de Bock GH. The Effect of Risk-Reducing Salpingo-Oophorectomy on Breast Cancer Incidence and Histopathological Features in Women with a BRCA1 or BRCA2 Germline Pathogenic Variant. *Cancers* 2023;**15**(7) doi: 10.3390/cancers15072095[published Online First.
